# Scaling up interventions for depression in sub-Saharan Africa: lessons from Zimbabwe

**DOI:** 10.1017/gmh.2016.8

**Published:** 2016-04-11

**Authors:** D. Chibanda, R. Verhey, E. Munetsi, S. Rusakaniko, F. Cowan, C. Lund

**Affiliations:** 1 Zimbabwe Aids Prevention Project, University of Zimbabwe, Community Medicine & Psychiatry, Harare, Zimbabwe; 2 Zimbabwe Aids Prevention Project, University of Zimbabwe, Community Medicine, Harare, Zimbabwe; 3 Centre for Sexual Health and HIV AIDS Research Zimbabwe, Adolescent and Adult Sexual Health, Harare, Zimbabwe; 4 University of Cape Town, Alan J Flisher Center for Public Mental Health, Department of Psychiatry and Mental Health, Cape Town, South Africa

**Keywords:** Common mental disorders, policy and systems, scale-up, stakeholder engagement

## Abstract

**Background:**

There is a dearth of information on how to scale-up evidence-based psychological interventions, particularly within the context of existing HIV programs. This paper describes a strategy for the scale-up of an intervention delivered by lay health workers (LHWs) to 60 primary health care facilities in Zimbabwe.

**Methods:**

A mixed methods approach was utilized as follows: (1) needs assessment using a semi-structured questionnaire to obtain information from nurses (*n* = 48) and focus group discussions with District Health Promoters (*n* = 12) to identify key priority areas; (2) skills assessment to identify core competencies and current gaps of LHWs (*n* = 300) employed in the 60 clinics; (3) consultation workshops (*n* = 2) with key stakeholders to determine referral pathways; and (4) in-depth interviews and consultations to determine funding mechanisms for the scale-up.

**Results:**

Five cross-cutting issues were identified as critical and needing to be addressed for a successful scale-up. These included: the lack of training in mental health, unavailability of psychiatric drugs, depleted clinical staff levels, unavailability of time for counseling, and poor and unreliable referral systems for people suffering with depression. Consensus was reached by stakeholders on supervision and support structure to address the cross-cutting issues described above and funding was successfully secured for the scale-up.

**Conclusion:**

Key requirements for success included early buy-in from key stakeholders, extensive consultation at each point of the scale-up journey, financial support both locally and externally, and a coherent sustainability plan endorsed by both government and private sectors.

## Introduction

There is growing evidence supporting the use of lay health workers (LHWs) through task-shifting (sometimes referred to as task sharing) to address the treatment gap for depression and other common mental disorders (CMD) in low and middle-income countries (Joshi *et al.* 2014). While this evidence seems relatively robust (van Ginneken *et al.* 2013), there is a dearth of well-documented examples of packages of care that have been taken to scale (Eaton *et al.* 2011).

Addressing critical knowledge gaps related to policy and systems that would encourage scaling up of evidence-based mental health interventions is needed (Tomlinson *et al.* 2009). A key strategy on how to achieve this includes sustained advocacy by diverse stakeholders, targeting multilateral agencies, donors, and governments (Lancet Global Mental Health Group *et al.* 2007). In addition the new role of psychiatrists and clinical psychologists should emphasize a public mental health approach, encompassing design and management of mental health initiatives at community level (Patel, 2009). Ideally, this new role should focus on providing support for task-sharing initiatives through, supervision, training, and the development of strategies for integrating and sustaining mental health care packages into existing programs such as HIV. Existing packages such as the mental health gap (mhGAP) (http://www.who.int/mental_health/mhgap/en/) could initially be used to empower and prepare clinicians for this new role.

Zimbabwe, with a population of 13 million people has eight psychiatrists and 16 clinical psychologists who are largely involved in clinical work in both tertiary and private facilities. In response to the high burden of depression in primary health care facilities, particularly among people living with HIV an intervention (The Friendship Bench) utilizing a task shifting approach was introduced in the city of Harare in 2006 (Chibanda *et al.* 2011). The Friendship Bench, an intervention delivered by trained and supervised LHWs provides basic cognitive behavior therapy with an emphasis on problem solving therapy (PST) (Mynors-Wallis, 2001). The intervention has been running as a pilot program in three primary care clinics for over 8 years with a focus on screening and treating depression and other CMD, locally referred to as *kufungisisa* (Patel *et al.* 1995), using locally validated tools (Patel *et al.* 1997). The intervention is delivered on a Bench (The Friendship Bench) in a discrete part of the clinic. The intervention consists of listing problems, identification of a single problem that the client wishes to focus on, brain storming, and developing a solution to that problem, which is specific and measurable (Chibanda *et al.* 2011). Clients are encouraged to go out and try the identified solution and discuss the outcomes of the initiative. There is an emphasis on encouraging LHWs to elicit solutions from clients, rather than provide advice for the clients on what the LHWs think should be done. An algorithm of care is used to decide on the flow of patients with a team consisting of clinical psychologists, mental health supervisors, and a psychiatrist providing the stepped care support to the LHWs (Chibanda *et al.* 2011; Chibanda *et al.* 2015*a*).

Recently, the Friendship Bench was evaluated through a cluster randomized controlled trial (RCT) which involved 24 primary care clinics (Chibanda *et al.* 2015*a*). Although preliminary results of the RCT showed promising impact the challenge facing the research team and key stakeholders was how to scale-up this evidence-based intervention to 60 existing primary health care clinics in the city. This article describes the process undertaken by key stakeholders to ensure that a scale-up plan, which was feasible and acceptable, was in place for commencement in October 2015 to be delivered in the 60 clinics. The approach we used is in keeping with the Medical Research Council Framework for the Evaluation of Complex Interventions (Craig *et al.* 2008), which indicates that the evaluation of the scale-up of a complex intervention requires multiple methods and an emphasis on process as well as outcome evaluations. The focus of this paper is on the process evaluation, and outcomes are reported elsewhere including the study protocol (Chibanda *et al*. 2015*a*).

## Method

A mixed methods approach was utilized to collect data from key sources to inform the team on an effective strategy for the scale-up. Four specific methods were used in a sequential manner, initially as part of formative work leading to a cluster RCT of the Friendship Bench described in the trial protocol (Chibanda *et al.* 2015*a*) and later as part of an ongoing process to develop an evidence-based strategy for scale-up. Triangulation of data was through an iterative process and review of existing records in the department of city health services. The activities were carried out as follows: (1) needs assessment using a semi-structured questionnaire to obtain information from nurses (*n* = 48) and focus group discussions (FGD) with all District Health Promoters (DHPOs) (*n* = 12) to identify key priority areas was carried out first, to inform the design of the trial and to identify key areas of focus for the scale-up strategy. All the participants for the needs assessment were employed by the city health department and had been identified as critical personnel for a successful scale-up by the director and nursing staff; (2) skills assessment to identify core competencies and current gaps of LHWs (*n* = 300) employed by city health services was used to inform the development of a training manual for both the cluster RCT and the scale-up strategy. All LHWs employed by the city health services were eligible for the skills assessment. Characteristics of these LHWs are described in Table 1; (3) consultation workshops (*n* = 2) with key stakeholders to determine referral pathways provided information leading to the development of an algorithm of care for the intervention arm of the RCT. Participants to the consultation workshops had been selected based on their current and previous work related to mental health in both the public and private sectors, including non-governmental organizations (NGOs); and (4) in-depth interviews and consultations to determine funding mechanisms for the scale-up was carried out after preliminary RCT results indicated that the intervention was superior to enhanced usual care. Participants to this component had participated in the consultation workshops described above.

(1) The needs assessment consisted of an initial visit to all 60 clinics in Harare by one of the authors (E. M.) and research assistants (*n* = 8) to establish what challenges clinics were facing with regard to integrating depression care packages into primary healthcare. Nurses from all the clinics were interviewed using an interview guide developed by the team aimed at establishing clinic priorities. Key issues highlighted by each clinic were compared and similar issues grouped together. The knowledge gained from the individual clinic visits was shared through two FGD with 12 DHPOs representing the 12 districts of Harare. The nominal group technique (NGT) was used for the first FGD. The NGT is used in situations where group consensus is needed and can include both health professionals and consumers, since it allows for the free exchange of opinions and the generation of ideas within a structured and non-hierarchical discussion forum (Allen *et al.* 2004). In this study the NGT commenced with each participant giving their views on integration of depression and CMD care packages into primary health care services. These views together with the solutions were then listed for each participant for all to see. The group then collectively ranked the solutions and the highest ranked was selected. Subsequent interaction utilized the Delphi technique (Lyons, 1982) where information was gathered primarily through electronic communication. Through the Delphi technique, emails were sent out to key stakeholders to obtain their views on how the scale-up could be realized. A summary of the responses was put together and sent out again for all to see allowing stakeholders to make changes to the summary until a group consensus was reached.
(2) Two assessments were conducted to determine current core competencies of 300 LHWs, based on previous experience on the Friendship Bench (Chibanda *et al.* 2011) and recommended core competencies developed through a meeting of stakeholders in Kampala, Uganda (Collins *et al.* 2015). A guideline of core competencies that were deemed necessary to deliver the Friendship Bench intervention included the following: minimum literacy equivalent to 10 years of education; ability to read and write, use a mobile phone, and send short message service (Chibanda *et al.* 2015*a*); and ability to recognize signs and symptoms of depression and CMD. All LHWs (*n* = 300) from the city health department were initially assessed for each of these components. After the first assessment, all those meeting initial competency criteria were further subjected to a face to face interview aimed at establishing their ability to recognize at least four signs and symptoms of depression and CMD such as poor sleep, being withdrawn, unable to function, feeling tearful, feeling run down, suicidal ideation or deliberate self-harm; communication skills; ability to show empathy and cultural competence; basic knowledge of the etiology of depression; ability to communicate with the community about depression (kufungisisa) based on the Kampala guidelines(Collins *et al.* 2015). From this assessment a final group of LHWs (*n* = 24) was selected and trained in the delivery of the PST intervention for the RCT (Chibanda *et al.* 2015*a*) and peer supervision. Those not selected were paired with the selected LHWs for peer support.
(3) Consultation workshops were conducted to finalize the referral pathway based on the protocol for the RCT of the Friendship Bench (Chibanda *et al.* 2015*a*). Through consultations with key stakeholders during a theory of change (ToC) workshop and a review of the literature (Annells *et al.* 2011; Collins *et al.* 2015; Noga *et al.* 2015) adjustments to the referral pathway were recommended. The ToC workshop focused on describing the causal pathway for the cluster RCT, looking at assumptions, barriers, indicators and interventions required to achieve program objectives. The full details of the ToC are described elsewhere (submitted International Journal of Mental Health Systems). Key adjustments to the referral pathway included the integration of a care package for mentally ill criminal offenders released back into the community from prisons. NGOs working within the prison services had expressed concern at the absence of a support system for discharged mentally ill offenders in Zimbabwe with conditions such as epilepsy, substance use disorders and psychosis. A final workshop with all stakeholders from the community, user groups, prison services, tertiary facilities and policy makers outlined key requirements of the referral pathway in the form of a flow diagram.
(4) In-depth interviews and consultations were conducted with collaborating partners from both local and international institutions and NGOs, to identify funding mechanisms for specific grant applications. The focus was to ensure availability of resources and funds for scale-up to commence by October 2015.

**Table 1.** Characteristics of 187 lay health workers employed by the City Health Department

Characteristic	Number (*n *= 187)	%
Years of education		
<10 years	23	10.3
≥10 years	164	87.7
Age		
<50 years	19	9.6
≥50 years	168	90.4
Mobile phone use		
Poor	7	3.7
Fair	4	2.1
Good	176	94.1
Writing skills		
Poor	6	3.2
Fair	10	5.3
Good	171	91.4
Reading skills		
Poor	6	3.
Fair	9	5
Good	171	93.
Ability to use SMS		
Poor	85	45.5
Fair	8	4.3
Good	94	51.
Knowledge of symptoms		
Good	145	77

SMS, short message service.

## Results

### Needs assessment

A final list of five cross-cutting issues was developed during the needs assessment. These included: lack of training in mental health, unavailability of psychiatric drugs, too few nursing staff, lack of time for counseling by nurses, and poor and unreliable referral systems for people suffering with depression and CMD. Addressing these cross-cutting issues was described as critical for a successful scale-up of the Friendship Bench.

Out of 48 nurses in the 60 primary care clinics, a total of 45 (93%) were available for interviews. Of these 39 (81%) had been working within city health services for over 15 years. They were predominantly female 41 (84%) with a mean age of 54 (s.d. 4.6). Of those interviewed 40(83%) indicated that they were too busy to provide structured psychotherapy to patients and lacked training in the use of anti-depressants. Only four (7%) had previously received training in mental health. A total of 42 (92%) acknowledged the need for mental health services for depression particularly among people living with HIV. A total of 39 (80%) indicated the need to provide a reliable supply of medication for depression. The need to have in place a clear referral system was mentioned by 40 (93%).

The above findings were later compared with findings from the FGD with 12 DHPOs who were responsible for overseeing activities at clinic level and supervision of LHWs. The DHPOs, using the NGT reached consensus to address four key issues: professional support and supervision, training, availability of medication, and referral pathways. These four areas were later endorsed by the nursing staff in a separate FGD as priority needs for a successful scale-up strategy. Professional support was the main priority as it was felt that without this they (nurses and DHPOs) would have major challenges to support LHWs or manage depression (kufungisisa) at clinic level. Psychiatrists and psychologists were needed to provide support and supervision as both DHPOs and nurses felt that suicidal and deliberate self-harm behavior required specialist skills. Training was highlighted as key to ensure that nurses and DHPOs were able to identify those who were severe and needing referral. Availability of medication such as amitriptyline, and fluoxetine including basic knowledge of appropriate dosing was needed. DHPOs supported the need to have a clear referral pathway between clinics and tertiary facilities, including prison services since mentally ill inmates were being released from prison with no clear discharge plan to the community.

### Competency assessment

The competency assessment of the existing 300 LHWs was carried out by district. There are 12 districts in Harare with each district having 14–25 LHWs. Of the 300 existing LHWs 230 (76%) were available for interviews. Of these a total of 187 (81%) fulfilled most of the initial criteria for core competency (Table 1). The 187 LHWs further received a face to face interview resulting in the best 24 (18%) being selected and trained for the RCT while the remaining 176 were paired with the 24 for peer support.

### Referral pathway and supervision support

**Fig. 1.**
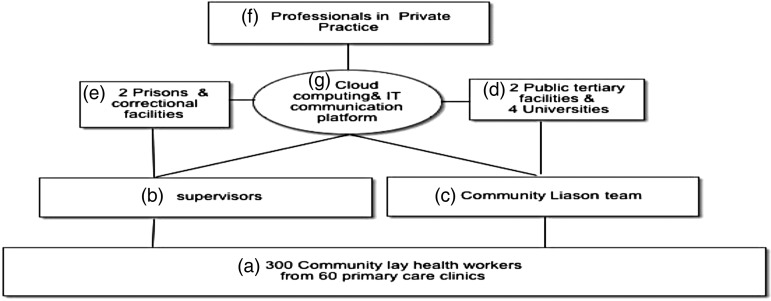
(*a*)–(*g*) Supervision and support structure.

The referral pathway was finalized through the development of a consolidated algorithm aimed at addressing all four priorities mentioned above. All those seen by the LHWs who were identified as having a ‘red flag’, which was defined as a score of 11 and above on the SSQ-14 or a ‘Yes’ response to question 11 on suicidal ideation on the SSQ-14 would immediately be referred to either the DHPO (supervisor) or to the community liaison team which consisted of a government doctor, nurse and clinical psychologist). A referral to the supervisor (DHPO) or community liaison team would result in the administration of the Patient Health Questionnaire (PHQ-9) to screen for depression. Scores above 10 on the PHQ-9 would be treated as confirmed depression and would be managed at local level with medication and the Friendship Bench or would result in further consultation through the information technology (IT) platform. This framework for the referral pathway was based on the developed referral plan for the RCT (Chibanda *et al.* 2015*a*). A supervision and support structure was developed to clarify the possible pathways of care for patients. The supervision and support strategy is illustrated in Fig. 1.

Providing support to the LHWs and the nursing staff was a critical component for a successful scale-up strategy modeled along earlier studies carried out in Zimbabwe (Abas *et al.* 1994; Chibanda *et al.* 2011; Chibanda *et al.* 2014*b*) and formative work leading to the trial protocol (Chibanda *et al.* 2015*a*). The pool of supervisors (Fig. 1*a*) consisted of the existing LHWs’ from the RCT who were designated as peer supervisors after the RCT (*n* = 24), their supervisors (Fig. 1*b*) DHPOs (*n* = 12) and nursing staff (*n* = 18). The main role of the supervisors was to receive all cases identified as ‘red flags’ by the LHWs (the remaining 274 who had not participated in the RCT) as described above. The supervisor would assess for further referral to (Fig. 1*d*) tertiary facility via (Fig. 1*c*) the out-reach team with possible support from the IT component used as a communication platform (Fig. 1*g*). The community liaison team (Fig. 1*c*) would visit selected clinics based on requests to provide support and manage difficult cases.

A key feature of the scale-up strategy is the referral of all incarcerated mentally ill offenders (Fig. 1*e*) back to the community via (Fig. 1*b, a*). The mentally ill offender upon discharge from a correctional facility is attached to a specific LHW who is responsible for follow up and monitoring, particularly through direct observed treatment strategies (observing patients physically take their medication) and to ensure that medications such as anti-epileptics and anti-psychotics are available at local clinic through regular communication with supervisors. The LHWs will also provide the PST approach to any former incarcerated mentally ill person presenting at the Friendship Bench, and requiring counseling while referring those that need care beyond their scope (Fig. 1*b*). Such an approach has been emphasized in the National Mental Health Policy. Public tertiary facilities and universities (Fig. 1*d*) would provide support through the formalized attachment programs to the Friendship Bench initiative and by providing an outreach team consisting of hospital staff and doctors. Clinical psychologists and psychiatrists in private practice (Fig. 1*f*) will provide support to the initiative via the IT platform through a virtual communication platform previously set up for the Friendship Bench during the RCT. The cloud computing component (Fig. 1*g*) will enable regular analysis of data and communication between the entire team but mainly between supervisors (Fig. 1*b*) and professionals in private practice (Fig. 1*f*) and tertiary facility level staff (Fig. 1*d*).

### Funding

From seven identified funding platforms and partnerships four applications were successful, of which one was local and three were international. One international agency Médecins sans Frontières (MSF) guaranteed availability of essential drugs for depression, epilepsy and psychotic illnesses and a focus on ensuring that capacity at tertiary and primary level was built through a series of workshops and ongoing teaching programs. This included support of an outreach team which will eventually be taken over by the local team employed by the government and city health services. The Wellcome Trust through a capacity building grant would enable the development of research excellence at masters, PhD, and postdoctoral level for the next 5 years, while the Grand Challenges Canada would support the completion of a cluster RCT and scale-up of the Friendship Bench. Locally, the city health department would fund all salaries for LHW, their supervisors, and medical staff working in tertiary facilities as part of building capacity within the Friendship Bench.

## Discussion

To our knowledge this is the first description of a strategy to scale-up an existing psychological intervention delivered by LHWs in sub-Saharan Africa. This scaled up intervention covers a population of over 1 million and is supported by the health authority through the provision of salaries and administrative support in all 60 clinics. Although emphasis has been on people living with HIV, this scale-up will include general health care services with a focus on depression and CMD, while emphasis on adherence to treatment for those on anti-depressant, anti-epileptic, anti-retroviral, and anti-psychotic medication will be part of the overall package provided within the PST approach. Earlier work in the region has justified such an approach by showing how task shifting could narrow the treatment gap at primary health care level in South Africa (Petersen *et al.* 2012; Lund *et al.* 2015).

Our paper highlights the steps taken to reach consensus on a scale-up strategy of the Friendship Bench which has been run as a pilot for over 8 years (Chibanda *et al.* 2011). Our development of a scale-up strategy focusing on LHWs has been a gradual process evolving over several years with initial stages aimed at defining indigenous concepts of CMD such as kufungisisa (Patel *et al.* 1997; Patel & Mann, 1997; Broadhead & Abas, 1998), developing culturally appropriate screening tools (Patel *et al.* 1997; Chibanda *et al.* 2009) carrying out systematic reviews of existing interventions (Chibanda *et al.* 2015*b*) and understanding key indigenous concepts required for effective delivery of the actual intervention (Chibanda submitted J Community Mental health), followed by testing the feasibility and acceptability of the intervention within an existing primary care setting (Chibanda *et al.* 2011) and through a RCT (Chibanda *et al.* 2015*a*). The steps we have followed over the years are in line with recommendations made on how to implement mental health care packages (Murray *et al.* 2014). Furthermore, as part of the scale-up we have introduced ongoing training sessions at the main tertiary hospital (Harare Central Hospital) as a continuous process of strengthening clinical competencies for both primary, secondary and tertiary level staff in line with recommendations for integrating mental health in primary care (Ventevogel, 2014).

This study will contribute to the body of growing knowledge on how to scale-up similar interventions, particularly within the context of HIV/AIDS.

Practical examples of scaling up interventions using LHWs have largely come from the field of HIV/AIDS where results have been promising (Zachariah R, 2009). While resource mobilization for fighting HIV/AIDS in sub-Saharan Africa has in the last 20 years been successful, the huge treatment gap and lack of political buy-in to address mental neurological and substance use disorders has been a major barrier in the scale-up of evidence-based interventions in mental health (Lancet Global Mental Health Group *et al.* 2007). Our approach has focused on integration into existing programs in order to improve mental health and non-mental health outcomes such as HIV/AIDS as previously recommended (Freeman *et al.* 2005; Chibanda *et al.* 2014*a*). Through a gradual and deliberate process of systematically engaging and involving key stakeholders, policymakers including the highest offices of the ministry of health and directorate of city health department, we have managed to provide evidence highlighting the importance of structured psychological interventions within HIV care settings and need for capacity building (Abas *et al.* 2014; Mangezi *et al.* 2014; Kidia *et al.* 2015).

Further achievements that have contributed to the scale-up strategy to be successfully completed include the partnerships with NGOs, local and international partners, which have led to the award of four major international grants in the last 6 years. These include the US Government's Medical Education Partnership Initiative (MEPI) linked award for Improving Mental Health Education and Research in Zimbabwe (IMHERZ http://www.nectar-uz.ac.zw/IMHERZ) which contributed to the establishment of an academic and research development plan for the country. The Grand Challenges Canada (GCC) mental health grant (0087-04) was built onto the MEPI award by focusing on strengthening community driven research. A Fogarty International Research grant (http://ghes.berkeley.edu) that focused on building the first author's research capacity, and most recently the African Mental Health Research Initiative (AMARI) grant awarded by the Wellcome Trust as part of its DELTAS scheme which aims to **D**evelop **E**xcellence In **L**eadership **T**raining And **S**cience, and the GCC transition to scale grant (0763-05).

These funding opportunities have enabled capacity building at several levels including university, private practice, and community.

A key and novel feature of our scale-up strategy is the inclusion of the private sector through the involvement of professionals in private practice as recommended by the Zimbabwe National Mental Health Policy. By enabling professionals to provide supervision and support through the use of computer tablets, which are virtually connected to LHWs at community level, we have managed to widen our support strategy thereby increasing the likelihood of sustainability of the initiative.

The Friendship Bench model has managed to function in three pilot sites for over 8 years with minimal funding, thus increasing the likelihood of sustainability once scaled up (Chibanda *et al.* 2011). Factors that have contributed to this include the integration of the intervention into existing systems within the city health services, the use of staff employed by the city health services, and the inclusion of tertiary facilities working under the Ministry of Health through clear referral pathways. These clear referral pathways include triaging clients based on locally validated tools with some being referred directly to higher level care while those scoring below the threshold for Friendship Bench being referred to alternative agencies (Chibanda *et al.* 2011; Chibanda *et al.* 2015*a*). As part of the scale-up, an international NGO (MSF) will focus on capacity building in the tertiary facilities including decongestion of the prison services through the expedited release of the mentally ill offenders back into the community.

Key lessons learnt from this scale-up action are the importance of early engagement with key stakeholders from government, NGOs, private sectors, and academic and research collaborating partners from external institutions both in Africa and beyond. The scale-up of the Friendship Bench has further been made possible through the creation of a core Friendship Bench team consisting of local and external individuals who have continued to provide mentorship and guidance even in the absence of external funding. The approach taken for this scale-up is in line with recommended strategies for avoiding the creation of narrow biomedical approaches to mental health care, such as the use of task shifting, use of brief psychological interventions, community-based recovery, and engaging communities as partners (Ventevogel, 2014). In the absence of well-documented examples of services that have been taken to scale (Eaton *et al.* 2011), the Friendship Bench could contribute to the new body of knowledge on scaling up mental health interventions. While similar scaled up programmes have been described in Ghana (Agyapong *et al.* 2015*a*, *b*), our initiative includes LHWs with minimal education.

There are a number of limitations to this scale-up strategy. We focused on literacy as a core requirement for LHWs, but there may be a number of other core competencies, which are essential and need to be assessed, such as the ability to show empathy, communicate effectively, and summarize clients’ presenting problems. While most of the LHWs met our inclusion criteria we had to find ways of integrating those who did not meet our criteria – these were paired with more qualified peers and then incorporated into home visit teams and the income generating groups which did not follow rigid structure for delivery as compared with the PST approach used on the Bench. This approach based on lessons learnt from earlier pilot work has been effective (Chibanda *et al*. 2011). However, further research is needed to evaluate scaling up of task sharing approaches using less strict inclusion criteria for LHWs, to mimic likely dilution of interventions in real world scale-ups.

Furthermore, we acknowledge that many problems of depressed mood may have their origins in broader social and economic conditions, which may be beyond what the health system can address.

The exclusion of traditional and faith healers in the scale-up plan is a further limitation. There is need to consider their role in further expansion of this initiative as they are often consulted in African communities (McInnis & Merajver, 2011). The unstable social and economic environment that is prevalent in Zimbabwe despite current support from the highest offices within both the Ministry of Health and City Health Department, could affect sustainability of the initiative. It will be important for the key drivers of the Friendship Bench (D. C., R. V., E. M.) to continue with advocacy initiatives. It is encouraging to note that the Friendship Bench first started when the socio-economic environment in Zimbabwe was at its' lowest point marked by an inflation rate of over 1000% with no support from policy makers. Despite these challenges community enthusiasm managed to sustain the initiative. It is this community commitment that may hold the future of not just the Friendship Bench but for other similar interventions across sub-Saharan Africa.

## Conclusion

This stepped care intervention embedded in an existing health care system could be a practical model worth replicating in similar settings across the region particularly where LHWs are part of an existing health care system. Key requirements for success include early buy-in from key stakeholders, extensive consultation at each point of the journey, financial support both locally and externally, and a coherent sustainability plan which is endorsed by both government and private sectors.
